# Recurrent Ischemic Stroke in a Patient with Atrial Myxoma: A Case Report

**DOI:** 10.31729/jnma.6693

**Published:** 2022-11-30

**Authors:** Gentle Sunder Shrestha, Ankit Rimal, Shubha Kalyan Shrestha, Pramesh Sunder Shrestha, Subhash Prasad Acharyai

**Affiliations:** 1 Department of Critical Care Medicine, Tribhuvan University Teaching Hospital, Kathmandu, Nepal; 2 Department of Anaesthesiology and Intensive Care, Bir Hospital, Kathmandu, Nepal

**Keywords:** *cardiac tumour*, *myxoma*, *cardiac surgery*, *ischemic stroke*

## Abstract

Cardiac myxoma is an infrequent but curable cause of ischemic stroke. There are no guidelines addressing the timing of surgery to excise the tumour or for the use of thrombolysis or thrombectomy. We present a case with an ischemic stroke which was diagnosed to have atrial myxoma. She was planned for surgical excision of the tumour but suffered from a second ischemic stroke while awaiting surgery. This article aims to highlight vital aspects of this rare phenomenon and discuss the prospects of the timing of surgery and neurosurgical intervention. The importance of a proper cardiac evaluation in all cases of stroke is highlighted.

## INTRODUCTION

Cardiac myxoma, although rare, is the most common cardiac tumour and can present with ischemic stroke.^1^ It is an important aetiology for stroke in the young.^2^ The emboli can be a dislodged thrombus or a tumour fragment. Although intravenous and intra-arterial thrombolysis and thrombectomy are the treatment options, surgical excision of the myxoma remains the mainstay of treatment.^1,3^ While there are no established guidelines as to when myxoma excision should be done following a neurological event, management should be individualized with consideration to the severity of the stroke and underlying medical condition. We have discussed a case of cardiac myxoma with the unusual presentation of recurrent bilateral ischemic stroke.

## CASE REPORT

A 37-year-old woman visited the emergency room with sudden and brief loss of consciousness, with weakness in the right side of her body. She had no comorbidities in the past. Examination revealed blood pressure of 130/80 mm of Hg, pulse rate of 76/min, temperature of 98.6°F, respiratory rate of 15/min, and SpO_2_ of 96% in room air. The patient had a Glasgow Coma Scale (GCS) of E4V1M6 with global aphasia. Pupils were round, regular, and reactive. Plantar reflex was downgoing on the left and upgoing on the right. Motor power was one-fifth in the right upper and lower limbs. Cardiac examination revealed a 4/6 mid-diastolic murmur at the apex. Pulmonary and abdominal examinations were normal.

On admission laboratory parameters revealed a haemoglobin of 11.9 gm% and a total white cell count of 9800/cumm. Platelet count was 447,000/cumm and Prothrombin Time (PT) was 13 sec with International Normalized Ratio (INR) 1. Urinalysis, blood sugar, electrolytes, renal and liver function tests were within normal limits. Chest roentgenogram appeared normal. Electrocardiogram (ECG) showed sinus rhythm. Plain Computed Tomography (CT) scan of the head revealed a left-sided ischemic stroke involving the middle cerebral artery territory. Transthoracic echocardiography revealed a heterogeneous, irregular mass measuring 2.6 x 4.8 cm arising from interatrial septum in the left atrium, near the fossa ovalis, and prolapsing towards left ventricle, suggestive of left atrial myxoma (Figure 1). After these relevant findings, the patient was planned for surgical excision and subsequent histopathological confirmation of the suspected tumour. Left ventricular systolic and diastolic functions were normal. Doppler study showed normal carotid and vertebral arteries.

**Figure 1:**
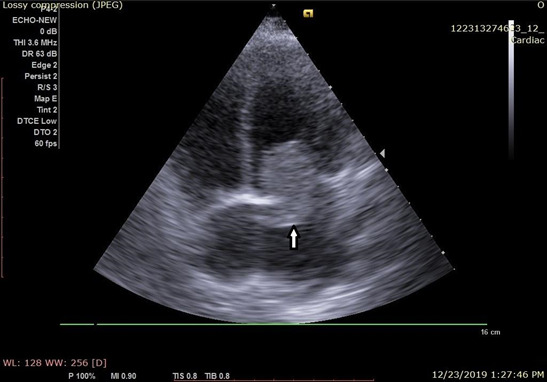
Transthoracic echocardiography revealed a mass arising from inter-atrial septum in the left atrium, and prolapsing towards left ventricle, suggestive of left atrial myxoma (indicated by arrow).

Three weeks later, while in the hospital awaiting surgical excision of the myxoma, her GCS dropped to E2V1M3. She was intubated and a repeat CT scan showed a new ischemic stroke on the right parietal region (Figure 2).

**Figure 1:**
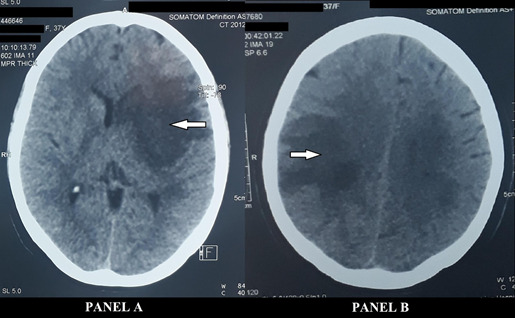
Panel A) Left sided ischemic stroke involving middle cerebral artery territory, Panel B) New infarction in right parietal region (both indicated by arrows).

Surgical decompression was deferred in view of reluctance on the part of the patient's family citing poor outcome. Supportive care was continued. Tracheostomy was performed on the 11^th^ day followed by gradual weaning. The patient was shifted out of the Intensive Care Unit to the ward with GCS of E4VTM5 with oxygenation maintained in room air. Her stay in the hospital was uneventful thereafter and she was discharged on request of the family in view of her static condition. The family was advised for follow up the next week. The patient did not come for the follow up and on inquiry, she had expired at home two weeks following discharge from hospital.

## DISCUSSION

Cardiac tumors are uncommon with a frequency of approximately 0.02% based on data from a large autopsy series.^4^ Cardiac myxomas are the most common among primary cardiac tumors and are usually amenable to surgical resection.^5^ Ischemic stroke due to a cardiac myxoma is commonly seen in the younger population and with female preponderance.^1,5^ Diagnostic triad include constitutional symptoms, embolic phenomena and features of obstruction.^6^ Obstructive symptoms manifest as dyspnea, orthopnea, edema, giddiness, syncope and sometimes as sudden cardiac death. We observed two separate embolic events in our patient as evidenced by clinical symptoms and CT findings. A study conducted in Germany suggested that the risk of recurrent stroke is increased with time elapsing between the neurological event and the excision of the tumor.^1^

Surgical resection is curative for most patients and would prevent the risk of subsequent cerebral or systemic embolization.^7^ Treatment during the acute phase of ischemic stroke is a subject for contention. Various modalities employed are intravenous thrombolysis, intra arterial thrombolysis and mechanical thrombectomy. In the published case series, thrombolysis has achieved mixed results with improvement seen in majority of patients.^8^ Despite anticoagulation therapy, there was a recurrence of cerebral embolisation in 2 of 5 patients in a case series.^7^ Anticoagulation would be of benefit in reducing thrombus embolisation but its usefulness for the prevention of tumour emboli seems unjustified. A study conducted in Germany concluded that these therapies were not alternatives to surgical excision since cerebrovascular events occurred in 46% of the patients despite their use.^1^ Furthermore, this recurrence risk is independent of the size of the myxoma and associated cardiovascular risks and is dependent, rather, on tumour friability. In addition, the risk of a neurological event recurring grows with increasing time intervals between the index neurosurgical event and surgical excision of the myxoma.^1^ In another study, the benefits of intravenous thrombolysis in patients who present after 4.5 hours have been argued.^9^ Our patient presented initially beyond this window. Thrombolysis was not attempted during the second event as the family was unwilling to proceed with it.

Although surgical resection of the myxoma is curative in that it removes the sources of the emboli, there is no consensus on how long to wait before open heart surgery after an acute neurologic event. A 4-week delay of the surgical resection has been suggested in a study, citing the risk of intracerebral haemorrhage following cerebral infarction.^10^ Cerebral autoregulation is impaired following stroke and the risks of bleeding following systemic anticoagulation required for open heart surgery is immensely high.^11^ In a study conducted in the US, the beneficial effects seen with early surgery in patients with TIA or retinal ischemia who do not have acute intracerebral infarcts have been described.^12^ A multidisciplinary evaluation with a neurologist, haematologist and cardiac surgeon, aimed at individualized management for patients with cerebral infarcts is prudent.

Our case highlights the need for a thorough cardiac evaluation of all patients with stroke. Lytic therapies seem of benefit within a narrow window when the embolus is a dislodged thrombus or a propagated thrombus, but would not prevent the recurrent stroke. Early surgery resection of myxoma is logical in the absence of acute infarcts. In a patient like ours, the balance between the risk of early surgery and the adverse effects of delayed surgery need to be weighed with the multidisciplinary approach to maximize neurological recovery and minimize complications.
